# *FcER1*: A Novel Molecule Implicated in the Progression of Human Diabetic Kidney Disease

**DOI:** 10.3389/fimmu.2021.769972

**Published:** 2021-12-01

**Authors:** Swastika Sur, Mark Nguyen, Patrick Boada, Tara K. Sigdel, Hans Sollinger, Minnie M. Sarwal

**Affiliations:** ^1^ Division of Transplant Surgery, University of California San Francisco, San Francisco, CA, United States; ^2^ Department of Surgery, University of Wisconsin School of Medicine and Public Health, Madison, WI, United States

**Keywords:** diabetes, DKD, FcER1, chronic kidney disease, mast cells

## Abstract

Diabetic kidney disease (DKD) is a key microvascular complication of diabetes, with few therapies for targeting renal disease pathogenesis and progression. We performed transcriptional and protein studies on 103 unique blood and kidney tissue samples from patients with and without diabetes to understand the pathophysiology of DKD injury and its progression. The study was based on the use of 3 unique patient cohorts: peripheral blood mononuclear cell (PBMC) transcriptional studies were conducted on 30 patients with DKD with advancing kidney injury; Gene Expression Omnibus (GEO) data was downloaded, containing transcriptional measures from 51 microdissected glomerulous from patients with DKD. Additionally, 12 independent kidney tissue sections from patients with or without DKD were used for validation of target genes in diabetic kidney injury by kidney tissue immunohistochemistry and immunofluorescence. PBMC DKD transcriptional analysis, identified 853 genes (p < 0.05) with increasing expression with progression of albuminuria and kidney injury in patients with diabetes. GEO data was downloaded, normalized, and analyzed for significantly changed genes. Of the 325 significantly up regulated genes in DKD glomerulous (p < 0.05), 28 overlapped in PBMC and diabetic kidney, with perturbed FcER1 signaling as a significantly enriched canonical pathway. *FcER1* was validated to be significantly increased in advanced DKD, where it was also seen to be specifically co-expressed in the kidney biopsy with tissue mast cells. In conclusion, we demonstrate how leveraging public and private human transcriptional datasets can discover and validate innate immunity and inflammation as key mechanistic pathways in DKD progression, and uncover *FcER1* as a putative new DKD target for rational drug design.

## Introduction

Systemic disease diabetes mellitus (DM) is characterized by an inability of the body to either produce or effectively respond to the glucose-regulating hormone, insulin. The International Diabetes Federation 2017 estimates that there are 425 million people (both diagnosed and undiagnosed) with diabetes in the world, which will reach 629 million people by 2045. The kidney is a highly vulnerable tissue in the diabetes milieu as the prevalence of end-stage renal disease (ESRD) is up to 10 times higher in people living with diabetes (1). Diabetic nephropathy or diabetic kidney disease (DKD) is a key microvascular complication of diabetes, classically identified by the presence of proteinuria (microalbuminuria in early stages and macroalbuminuria as DKD advances) in people with diabetes. However, increasing evidence has shown that a significant number of patients with type 2 DM may have decreased glomerular filtration rate (GFR) without significant albuminuria, known as non-albuminuric DKD (2). Progression of DKD is more likely to occur in patients who have long-standing diabetes, poor glycemic control, or associated morbidities such as hypertension or obesity. But the progression rate to kidney failure in people who are non proteinuric but living with diabetes is much lower than those who are proteinuric (3). The lifetime risk of DKD is roughly equivalent to type 1 (insulin-dependent, juvenile-onset) and type 2 (adult-onset) diabetes (4, 5). There are no identified therapies that can specifically target reversal or slow down the progression of DKD injury. Current DKD management involves general measures including, lifestyle modification, blood pressure control, glycemic control, plus the use of lipid-lowering drugs, albuminuria-reducing drugs, and treatment with sodium-glucose co-transporter 2 (SGLT2) inhibitors (6–11). Among people with diabetes, the development of DKD carries a higher mortality risk. A substantial proportion of people with DKD will have progressive loss of kidney function and will develop ESRD (12). Hence, there is a clear unmet need to understand the specific biological basis of kidney injury in diabetes, to understand pathways that contribute to progressive DKD injury, and develop specific DKD-targeted therapies that can reverse or slow down the unrelenting progression of DKD and, improve both the quality and quantity of life for this patient group.

The development and progression of DKD is thought to involve a combination of hemodynamic, metabolic, ischemic, and inflammatory factors (13–16). However, the exact mechanisms of kidney injury in a hyperglycemic milieu, with underlying genetic and racial risk factors that may increase DKD disease risk, remain to be elucidated. The predominant structural changes include mesangial expansion, glomerular basement membrane thickening, podocyte injury, and, ultimately, glomerular sclerosis (17). Albuminuria and progressive chronic kidney disease (CKD) are major clinical manifestations of DKD (18). Although, classic lesions of diabetic nephropathy is similar in both type 1 and type 2 diabetes, however, renal lesions are more heterogeneous in patients with type 2 diabetes with some patients developing more advanced vascular or chronic interstitial lesions than diabetic glomerulopathy (19, 20). The principal biomarkers presently used to predict DKD progressions are albuminuria and estimated glomerular filtration rate (eGFR) (21). However, not all cases of classical DKD are accompanied by increases in albuminuria, which reduces the value of this biomarker, particularly in early DKD (22–24). Optimizing the management of diabetes can reduce the rate of kidney function decline, but with a lack of clarity on DKD pathogenesis, specific DKD therapies have not been developed to date (25).

We hypothesized that innate immunity and inflammation may play an important mechanistic role in DKD progression. Our study is designed to evaluate such perturbations in the kidney tissue and circulating blood of people with diabetes, and then interrogates the expression of specific markers in the biosamples with the increased severity of diabetic kidney damage and dysfunction. The hypothesis stems from the fact that dendritic cells, mast cells, and macrophages have all been reported to infiltrate diabetic kidneys (15, 26). A population study of diabetes revealed a positive correlation between plasma IgE and diabetes or prediabetes (27). Increased levels of eosinophilia positively correlated with worsening stages of CKD and DKD (28), thus providing evidence of dysregulated innate immunity. However, this is not an invariant finding in all people living with diabetes. Histological findings are consistent with apparent observations of mast cells and eosinophils in kidney tissues from DKD.

**Figure 1:**
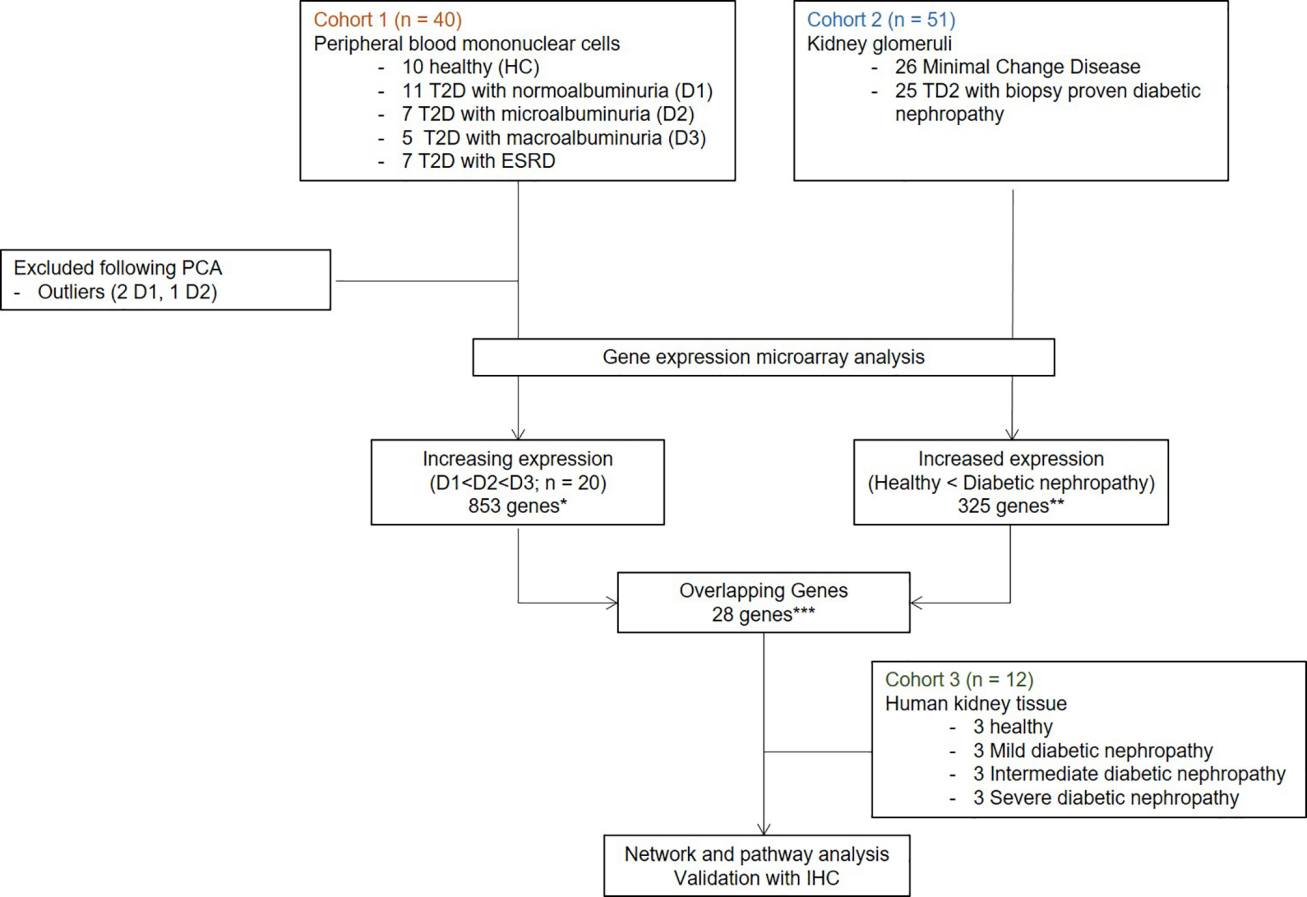
Overall study design and workflow. Transcriptome profiling with gene microarray performed on PBMCs from demographically matched sets of patients at different stages of DKDs (Cohort1) integrated with the publicly available microarray experiments on four microdissected glomeruli of patients with DKD (Cohort 2). Customized bioinformatics allowed for the selection of genes that correlated with DKD severity in both data sets by hypergeometric enrichment. Network and pathway analysis was performed with significant overlapping genes. One target with the most significant expression with DKD severity and the associated cell type was validated in an independent set of DKD samples (Cohort 3). IHC and IF for protein product performed on human kidney tissue. T2D, Type 2 diabetes; HC, healthy control; D1, normoalbuminuria; D2, microalbuminuria; D3, macroalbuminuria; IHC, immunohistochemistry; IF, immunofluorescence.

In this paper, we carefully mapped three independent, non-overlapping cohorts of individuals with or without diabetes, to identify genes that might play a crucial role in the development and progression of DKD. We performed an unbiased discovery analysis of gene expression changes in peripheral blood mononuclear cells (PBMC) collected from individuals with varying stages of DKD in Cohort 1. Analysis of genes in Cohort 1 was focused on evaluating pathways that may play a role in the proinflammatory component of DKD injury, and are also impacted by the advanced stages of kidney injury **(** **Table 1**). With the assumption that infiltration of leukocytes in the diabetic kidney may impact the severity of glomerular disease progression in diabetes, in Cohort 2 we performed unbiased discovery of transcriptional changes in the glomerulus of DKD tissue. We aimed to find enrichment of DKD specific overlapping genes between the two cohorts, which can then be used as a biomarker for DKD. Finally, in Cohort 3, we independently validated the protein expression and localization of the most enriched gene in renal tissue samples from varying stages of DKD, assessing if genes identified from Cohort 1 and 2 could be localized in DKD tissue. **Figure 1** summarizes the overall study design.

**Table 1:** Demographic information for Cohort 1.

Main Clinical Variables	Clinical Phenotypes				
	HC, n = 10	D1, n = 11	D2, n = 7	D3, n = 5	ESRD, n = 7
Age (years ± SD)*	36.6 ± 10.5	58.8 ± 6.4	61.4 ± 9.3	63.4 ± 4.2	57.4 ± 11.6
Sex (F/M)	7/3	7/4	4/3	1/4	4/3
Ethnicity	0/10	0/11	1/6	0/5	1/6
(Hispanic/Non-Hispanic)					
sCr (mg/dL ± SD)*	0.9 ± 0.1	0.9 ± 0.2	1.1 ± 0.3	1.5 ± 0.4	4.7 ± 2.4
eGFR (mL/min/1.73 m^2^ ± SD)*	82 ± 17.0	76.7 ± 19.5	65.3 ± 21.2	53.7 ± 22.1	20.8 ± 10.4
Albuminuria (mg/day ± SD)*	–	12.4 ± 7.2	89.3 ± 76.4	738.2 ± 248.25	–
Duration of Diabetes (years ± SD)	–	12.0 ± 6.7	12.7 ± 8.2	16.6 ± 5.9	15.4 ± 7.9
BMI (kg/m^2^ ± SD)	25.8 ± 6.7	35.0 ± 6.5	39.6 ± 7.8	32.0 ± 2.0	34.7 ± 2.7
SBP (mmHg ± SD)	124.4 ± 26.9	119.8 ± 11.0	120.6 ± 10.8	118.0 ± 21.3	135.3 ± 14.9
DBP (mmHg ± SD)	74.5 ± 14.9	68.6 ± 6.9	61.4 ± 5.9	62.8 ± 7.8	68.2 ± 8.3
HgA1c (nmol/mL/min ± SD)	–	8.7 ± 1.7	7.7 ± 1.3	8.1 ± 1.0	7.8 ± 1.5
Total Cholesterol (mg/dL ± SD)	187 ± 15.0	157.8 ± 30.4	150.1 ± 14.4	160.6 ± 35.0	124.3 ± 19.3
Insulin Therapy	0/10 (0%)	9/11 (82%)	5/7 (74%)	5/5 (100%)	5/7 (74%)
ACEi/ARB Therapy	0/10 (0%)	11/11 (100%)	7/7 (100%)	5/5 (100%)	5/7 (74%)

*p < 0.05. SD, Standard Deviation; HC, Healthy controls; D1, diabetes with normoalbuminuria; D2, diabetes with microalbuminuria; D3, diabetes with macroalbuminuria; sCr, serum creatinine; eGFR, estimated glomerular filtration rate; BMI, body mass index; SBP, systolic blood pressure; DBP, diastolic blood pressure; HgA1c, Hemoglobin A1c; ACEi, angiotensin converting enzyme inhibitor; ARB, angiotensin II receptor blocker.

## Methods

### Study Design and Samples

This project benefits from a unique study design, utilizing analysis of transcriptional data from 103 unique samples, from 2 different tissue sources: PBMC and kidney tissue. In addition to evaluating common gene expression signatures in DKD across both tissue sources, we also designed the study to evaluate the impact of transcriptional changes with the increasing severity of DKD injury. This study is divided into three cohorts, microarray gene expression analysis from PBMCs and kidney glomerulous (publicly available datasets) forms cohort 1 and 2, respectively, which was used for unbiased discovery of genes that correlated with DKD severity. Finally, in cohort 3, we performed *in situ* validation of the target gene expression in the DKD kidney (**Figure 1** **)** *in situ*.

Cohort 1 consisted of transcriptional profiling of PBMC from 30 unique DKD patients with different stages of CKD and 10 normal healthy controls without diabetes or kidney damage (no proteinuria and eGFR > 90 ml/min/1.73m^2^) (29). The 30 patients with diabetes have advancing stages of renal injury, as mapped by proteinuria and eGFR: 11 had eGFR > 90 ml/min/1.73m^2^ and without any microalbuminuria, 7 had eGFR > 90 ml/min/1.73m^2^, with microalbuminuria, 5 patients had eGFR between 60-90 ml/min/1.73m^2^, with macroalbuminuria, and 7 patients had macroalbuminuria and eGFR < 60 ml/min/1.73m^2^. DM patients were on insulin therapy. Demographic and clinical details are provided in **Table 1**. All blood samples were collected between November 2011 and August 2012 from a single academic center (University of Wisconsin). Written informed consent was obtained from all the participants, and the study was approved by the institutional review boards of the University of Wisconsin and the University of California, San Francisco.

Cohort 2 consisted of 51 unique patients where glomerulous had been microdissected and transcriptionally profiled (GSE1009, GSE47183, and GSE30528) from either patients with DKD (n=25) or without DKD controls (minimal change disease; n=26). Diabetic nephropathy is characterized by diffuse or nodular glomerulosclerosis, afferent and efferent hyaline arteriolosclerosis, and tubulointerstitial fibrosis and atrophy (30). In this study, we focused on glomerular genes that may contribute to glomerulosclerosis, which is the typical initial clinical manifestations of DKD. Since the hemodynamic phenotype in early diabetes is characterized by glomerular hyperfiltration which has been associated with progressive diabetic nephropathy, we selected genes enriched with expression in the kidney glomerulus for separations of the initial cohorts, as diabetes is a pathology that primarily results in changes in the kidney filtration unit. These smaller sub-compartment gene set differences allow for a greater understanding of specific changes in the kidney glomerulus in DKD injury, which eventually is associated with progressive diabetic nephropathy.

Cohort 3 consisted of 12 unique kidney tissue samples that includes, 3 patients without diabetes who had normal renal tissue resection at nephrectomy for renal tumor removal and 9 DKD patients with renal biopsies, with varying grades of CKD: 3 with eGFR > 90, 3 with eGFR 60-90 and 3 with eGFR < 60 ml/min/1.73m^2^. Cohort 3 served as the independent validation set for DKD specific renal tissue gene expression, as mined from an overlap of genes from Cohorts 1 and 2.

### PBMC Collection and RNA Extraction

Total RNA extracted from PBMC by the RNeasy mini kit (Qiagen); RNA concentration measured by NanoDrop^®^ ND-1000 (NanoDrop Technologies) and RNA integrity assessed by the Agilent 2100 Bioanalyzer using RNA Nano Chips (Agilent Technologies). RNA was stored in RNase-free water at −80°C until sample preparation for transcriptional analysis- unbiased discovery was done by microarrays (Agilent Technologies), and validation was done for target genes by quantitative reverse transcriptase-polymerase chain reaction (qRT-PCR).

### PBMC Microarray Hybridization

Complementary DNA (cDNA) was prepared by reverse transcribing the total RNA using T7-promoter primer and MMLV reverse transcriptase; 100 ng of total RNA processed by the Agilent LIRAK PLUS, two-color Low RNA input Linear Amplification method. cDNA was transcribed into complementary RNA (cRNA) and fluorescently labeled by incorporation of cyanine Cy5-CTP and Cy3-CTP. After purification, using the RNeasy mini kit (Qiagen), cRNA yield and Cy incorporation efficiency were determined using a NanoDrop Spectrophotometer (NanoDrop Technologies). Equal amounts of the diseased and reference control sample (825 ng) were competitively hybridized onto Agilent Whole Human Genome 4× 44K 60mer oligonucleotide arrays (G4112F, Agilent Technologies). Arrays were scanned on an Agilent scanner and processed by the Agilent Feature Extraction Software. Microarray data was uploaded to Gene Expression Omnibus (GEO) (Accession number GSE142153; https://www.ncbi.nlm.nih.gov/geo/query/acc.cgi?acc=GSE142153).

### Immunohistochemistry

Histological analysis of target gene expression was performed *via* immunohistochemistry (IHC) in human kidney tissue (Cohort 3). Formalin-fixed paraffin-embedded (FFPE) sections of 5 µm were stained with mouse monoclonal antibodies against our target gene of interest. We purchased the primary FcER1 antibody (sc-390222) and secondary m-IgG Fc BP-HRP (sc-525409) from Santa Cruz Biotech (Dallas, TX) and used at a dilution of 1:100 with appropriate negative controls.

### Immunofluorescence

Immunofluorescence (IF) was performed on FFPE tissue sections. Paraffin blocks were deparaffinized and rehydrated with heat-induced epitope retrieval with citrate buffer (pH 6.0) at and blocked by 5% BSA. Kidney tissue sections were incubated overnight with primary human antibodies. The primary antibodies used was: Mast cell tryptase, CD68, and CD45 (DAKO). The secondary antibody used were: anti-mouse IgG FITC and anti-rabbit IgG Texas red (Vector Laboratories). Sections were mounted in Prolong gold antifade-DAPI aqueous mounting medium (Invitrogen USA) and visualized (20X) using Leica DCF450C bright field/fluorescence microscope. The presence of various antigens was quantified by measuring the intensity of respective immunocomplexes using Definiens Tissue Studio (Definiens, Germany).

### Data Analysis

**Figure 2:**
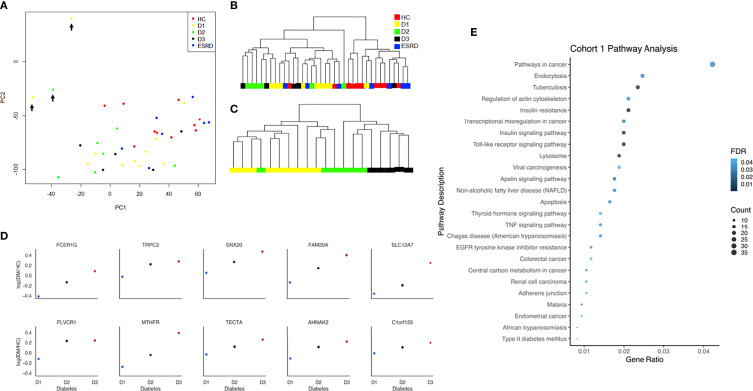
**(A)** Principal component analysis plot with all samples and unique genes from the microarray analysis performed on peripheral blood mononuclear cells (PBMCs) from patients at different stages of DKDs (Cohort 1). Outliers are marked with black arrows. **(B)** Dendrogram of unsupervised clustering for all samples of diabetes (D1, D2, D3), ESRD, and healthy controls without the outliers is based on the significant genes determined by ANOVA (p < 0.05). **(C)** The dendrogram of unsupervised clustering for samples with diabetes only without the outliers is based on the top 100 significant genes, whose expression increased with disease severity as determined by the JT test. **(D)** Facet grid plot of representative genes from cohort 1 with a positive correlation between its expression and degree of albuminuria shown as log fold change compared to control. Candidate genes were selected by taking the top ten statistically significant genes. The analysis demonstrates the higher expression of *FcER1* in more advanced stages of diabetes. **(E)** Dot-plot showing the top pathways in cohort 1. HC, Healthy control; D1, diabetes with normoalbuminuria; D2, diabetes with microalbuminuria; D3, diabetes with macroalbuminuria; ESRD, end stage renal disease.

All data analyses were run by using R version 4.04 and Python 3.8.8 (31, 32). The raw PBMC microarray data were processed using the Limma package in R and normalized within (loess) and between (aquantile) arrays (33). The levels of gene expression across different levels of proteinuria (i.e., normoalbuminuria, microalbuminuria, and macroalbuminuria) were measured and ranked by Jonckheere-Terpstra (JT). JT is a rank-based nonparametric method for testing differences between more than two groups. It was used to rank genes with increasing or decreasing expression in different levels of albuminuria (**Figure 2**). The JT test function of the SAGx package in R was implemented for this analysis (34). The Principal Component Analysis (PCA) plot showing unique genes from the microarray analysis performed on PBMCs from patients at different stages of DKDs has three outliers (**Figure 2A**), which are removed to plot the dendrogram of unsupervised clustering for all samples in **Figure 2B**. These 3 samples had poor sample quality with greater missing data which may account for their outlier distribution. Expecting some intra-cohort variability is expected in human phenotyping studies. Despite this, we show that the majority of patients in each cohort, as shown in **Figure 2B**, fall within groupings that are driven in a biologically meaningful manner. Data visualization was done with GraphPad. Significant trends in gene expression with the level of albuminuria were computationally determined. The facet grid plot (**Figure 2D**) was created by using Python 3.8.8. The analysis utilized the Pandas and Seaborn libraries (35, 36). Candidate genes were selected by taking the top ten statistically significant proteins.

**Figure 3:**
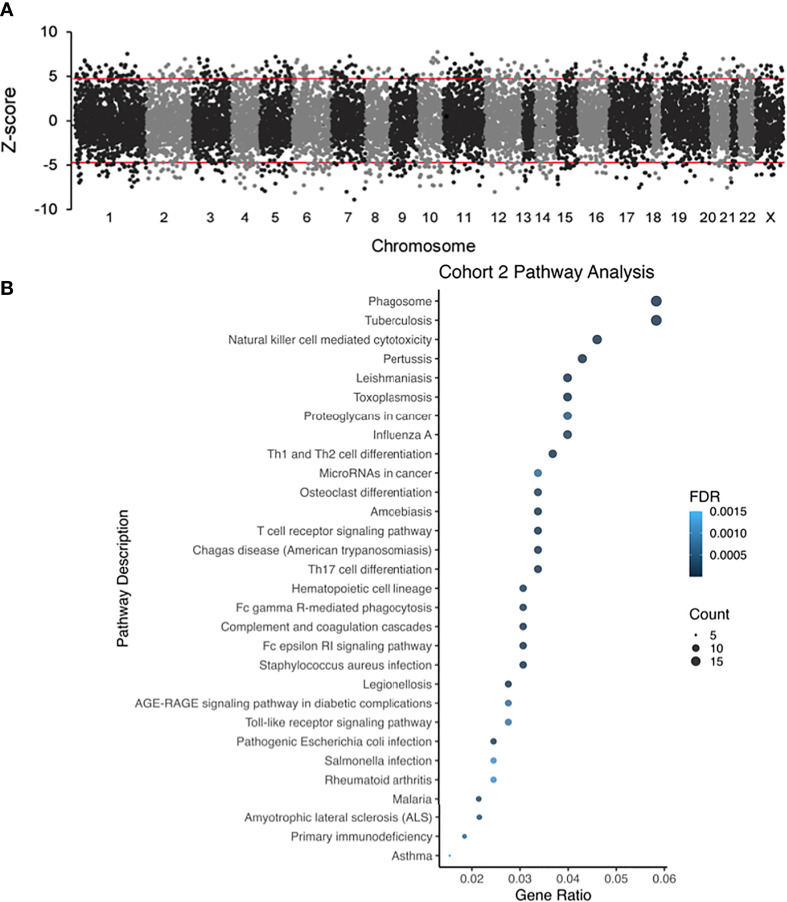
**(A)** Different glomerular microarray platforms in GEO were converted to Entrez Gene identifiers and analyzed by eGWAS (Cohort 2). Using a one-tailed T-test P values between DKD and controls were calculated, which were converted to Z-scores and meta-analysis was performed based on the weighted Z-method. Gene expression leading to a Z-score above 5 or below -5 was considered significantly up or downregulated. **(B)** Dot-plot showing the top pathways in cohort 2.

**Figure 4:**
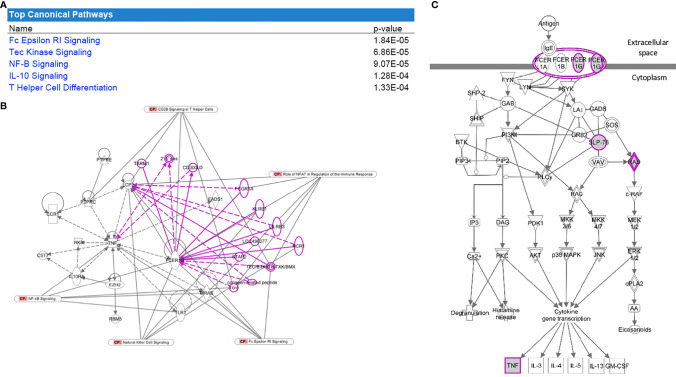
Pathway analysis of the overlapped gene expression that is common in cohorts 1 and 2. **(A)** Table with the top significant canonical pathways in the 28 genes were found to be significant for DKD and overlapping between both datasets. **(B)** FcER1 signaling was identified as the center node of the significantly enriched canonical pathway connecting most of the identified significant genes including *Tec Kinase, NF-B, IL-10*, and T Helper cell differentiation pathways. Nodes outside the gene-set with known interactions are displayed in pink. **(C)** It is the known signaling pathway of FcER1, where the proteins including FcER1, TNF, SLP, and Ras are also present in the common 28 genes list.

**Figure 5:**
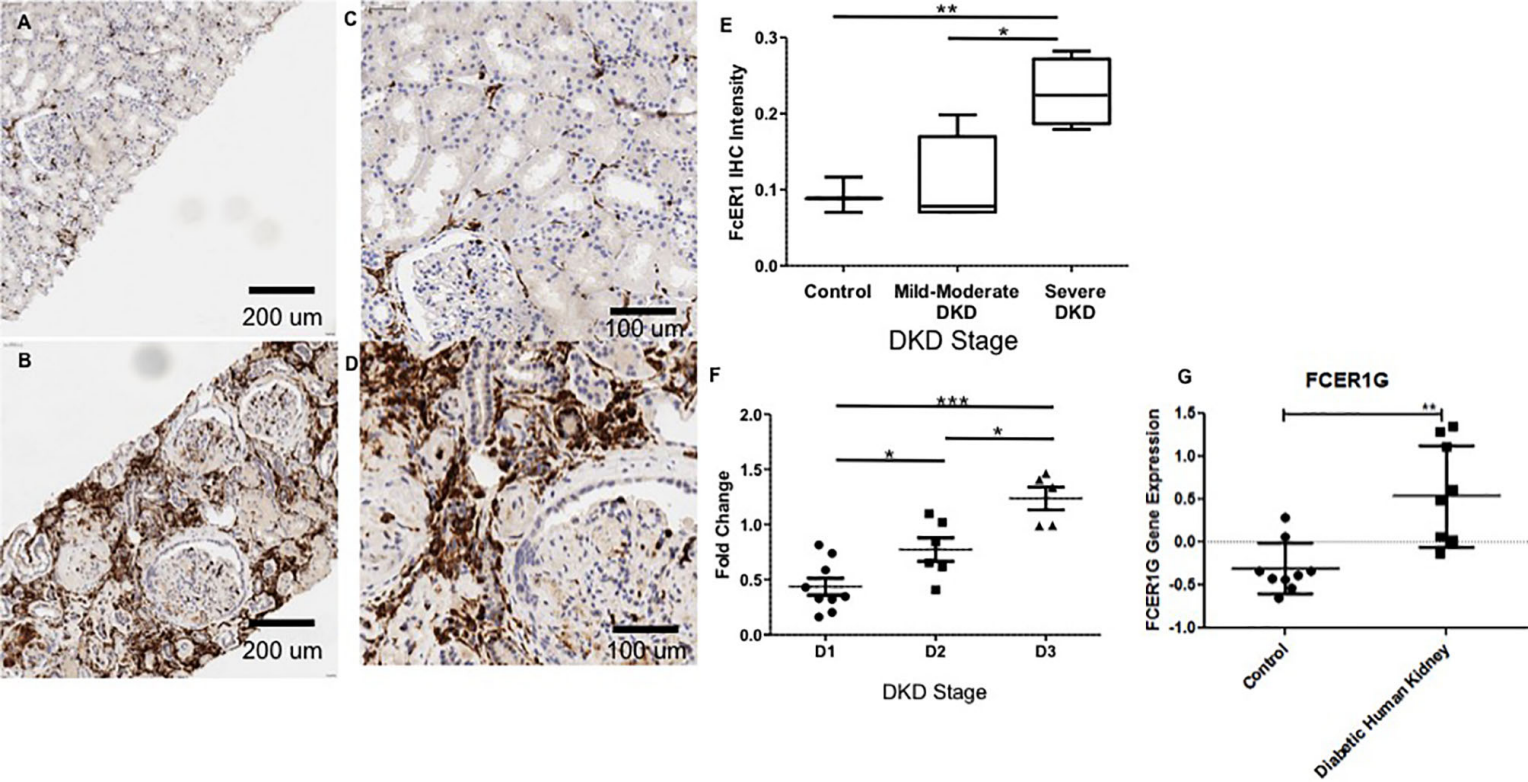
Representative Immunohistochemistry staining for *FcER1* in human kidney tissue. **(A)** Normal kidney at 20x. **(B)** Diabetic kidney at 20x. **(C)** Normal kidney at 40x. **(D)** Diabetic kidney at 40x. **(E)** The intensity of FcER1 staining in human kidney tissue. **(F)** Gene expression of *FcER1* from PBMCs at different stages of diabetic kidney disease. **(G)** Gene expression of *FcER1* in control and diabetic kidney disease; *p < 0.05, **p < 0.01, ***p < 0.001; DKD, diabetic kidney disease; D1, diabetes with normoalbuminuria; D2, diabetes with microalbuminuria; D3, diabetes with macroalbuminuria.

For analysis of Cohort 2, probe labels across different glomerular microarray platforms in GEO were converted to Entrez Gene identifiers with AILUN, and further analysis was performed by expression genome-wide analysis (eGWAS) (37). A one-tailed t-test was used to calculate P values between cases and controls. P values were converted to Z-scores and meta-analysis was performed based on the weighted Z-method. Expression leading to a Z-score above 5 or below -5 was considered significant. Manhattan plots were generated by using the qqman and qnorm R libraries (**Figure 3A**) (38, 39). Pathway analysis dot plots were constructed by using Pandas and ggplot2 (**Figures 2E**, **3B**) (40). For pathway analysis dot plots the FDR limit was set to <= 0.05. We calculated the likelihood of repeated differential expression of genes across the kidney samples with DKD and compared specific glomerular gene expression from Cohort 2 with completely independent PBMC gene expression data from patients’ with DKD in Cohort 1, to enrich for DKD specific biologically relevant gene expression signatures from different DKD tissue sources (**Table 2** and **Figure 4**). We used Ingenuity Pathway Analysis (IPA) (http://www.ingenuity.com/; QIAGEN, Redwood City, CA, USA) to connect a comprehensive list of genes potentially associated with the development of diabetes. IPA is a bioinformatic tool that connects a list of genes into a set of networks based on the Ingenuity Knowledge Base, which contains information on biomolecules (represented by nodes in the networks) and their relationships (represented by edges and arrows in the networks). In our study, we uploaded the overlapping genes between, cohorts 1 and 2, to IPA and performed the core analysis function to detect the signaling pathways that are potentially associated with diabetes. The resulting pathways, functions, and networks are scored based on the negative base-10 logarithm of the p value from a right-tailed Fisher’s exact test. The p values obtained using this test identify statistically significant enrichment of the focus genes in a given function, pathway, or network (41–43). Imaging in Cohort 3 was analyzed on Definiens Tissue Studio (Definiens, Germany) (**Figure 5**).

**Table 2:** List of genes with significantly increase expression in PBMCs and glomeruli.

Gene ID	Gene Description	p Value (eGWAS)	p Value (JT test; D1 < D2 < D3)
*FCER1G*	Fc fragment of IgE, high affinity I, receptor for; gamma polypeptide	3.345E-10	1.000E-04
*AHNAK2*	AHNAK nucleoprotein 2	2.201E-07	1.800E-03
*LCP2*	lymphocyte cytosolic protein 2 (SH2 domain containing leukocyte protein of 76kDa)	1.688E-08	3.500E-03
*ADAP2*	ArfGAP with dual PH domains 2	2.320E-07	6.400E-03
*MRAS*	muscle RAS oncogene homolog	6.823E-09	7.800E-03
*TLR1*	toll-like receptor 1	2.027E-07	1.130E-02
*SH3BGRL3*	SH3 domain binding glutamic acid-rich protein like 3	2.717E-07	1.130E-02
*C15orf39*	chromosome 15 open reading frame 39	2.932E-07	1.620E-02
*RHOC*	ras homolog gene family, member C	8.916E-08	1.930E-02
*DOCK10*	dedicator of cytokinesis 10	2.762E-07	1.930E-02
*TNF*	tumor necrosis factor	3.222E-08	2.280E-02
*PTPRE*	protein tyrosine phosphatase, receptor type, E	6.707E-08	2.280E-02
*STK10*	serine/threonine kinase 10	5.890E-08	3.150E-02
*CCR1*	chemokine (C-C motif) receptor 1	1.147E-07	3.150E-02
*RNF125*	ring finger protein 125	5.835E-10	3.150E-02
*EZH2*	enhancer of zeste homolog 2 (Drosophila)	5.788E-08	3.150E-02
*CPE*	carboxypeptidase E	1.137E-08	3.680E-02
*C9orf167*	chromosome 9 open reading frame 167	3.780E-07	3.680E-02
*PTPRC*	protein tyrosine phosphatase, receptor type, C	7.646E-08	3.680E-02
*TRIB3*	tribbles homolog 3 (Drosophila)	3.237E-07	3.680E-02
*IL10RA*	interleukin 10 receptor, alpha	6.394E-11	3.680E-02
*CENPM*	centromere protein M	2.518E-07	3.680E-02
*RBM5*	RNA binding motif protein 5	6.192E-08	3.680E-02
*C1orf77*	CDNA FLJ37911 fis, clone CTONG1000052	4.105E-07	4.290E-02
*HK3*	hexokinase 3 (white cell)	2.584E-07	4.290E-02
*CST7*	cystatin F (leukocystatin)	5.494E-07	4.960E-02
*PSTPIP2*	proline-serine-threonine phosphatase interacting protein 2	2.253E-07	4.960E-02
*EPB41L2*	erythrocyte membrane protein band 4.1-like 2	5.897E-08	4.960E-02

## Results

### Cohort 1: DKD Gene Expression in PBMCs Correlates With Disease Progression and Identifies *FcER1* as the Topmost Gene With the Highest Expression

Transcriptional analysis of PBMC samples from patients with progressive kidney injury and diabetes identified 853 genes that increased expression, and 1355 genes that decreased expression (JT test; p < 0.05), with the progression of albuminuria, and worsening of eGFR in patients with diabetes. The clustering of these samples before and after the JT test is shown in **Figures 2A**–**D**. In **Figure 2D**, candidate genes were selected by taking the top ten statistically significant genes. The analysis demonstrates the higher expression of *FcER1* in more advanced stages of diabetes. The toll-like receptor signaling pathway is the topmost enriched pathway with all the upregulated genes. *FcER1, ITGAX, SLC12A7* are a few of the top 30 significant genes, whose expression increased with the severity of diabetes (**Supplementary Table 1**). It is interesting to note that the PBMC transcriptome of health and ESRD cluster closer together (**Figures 2A, B**). The dot-plot in **Figure 2E** shows top pathways in cohort 1 that include Toll-like receptor, insulin-related, TNF, and type 2 DM signaling pathways.

### Cohort 2: Publicly Available Datasets Identify FcER1 Signaling as the Most Upregulated Pathway in DKD Glomerular Injury

Datasets in GEO were computationally interpreted eGWAS to identify differential gene expression associated with DKD glomerular injury (**Figure 3A**). We identified 325 significantly upregulated and 248 downregulated genes in DKD glomerulous. The dot-plot in **Figure 3B** shows top pathways in cohort 2 that includes, T-cell receptor, FcGR-mediated, FcER1-mediated, AGE-RAGE, and Toll-like receptor signaling pathways. Pathway enrichment analysis showed that the FcER1 signaling pathway is the most upregulated KEGG pathway and various types of immune responses (e.g.defense, positive regulation, activating signal transduction) are significantly high among all other biological processes. It was interesting to note that biological processes including kidney and nephron development are significantly downregulated.

### Overlapping Genes From Cohorts 1 and 2 That Correlate With DKD Severity Identified FcER1 Signaling as the Common Most Enriched Pathway

We interrogated overlapping increased specific gene expression from the meta-analysis of publicly available transcriptional data (cohort 2) with lab-generated PBMC transcriptional studies in DKD (cohort 1). Twenty-eight genes were found to be significant for DKD and overlapping between both datasets. Pathway analysis of these enriched genes with IPA revealed canonical pathways that are significantly overrepresented in this gene set are presented in **Figure 4A**. *FcER1G* was found to be the most significantly expressed gene dysregulated in DKD tissue showed increased expression in PBMC with increasing severity of DKD renal injury (**Table 2**). FcER1 signaling was also identified as one of the significantly enriched canonical pathways along with Tec Kinase, NF-B, IL-10, and T Helper cell differentiation pathways. **Figures 4B** shows that *FcER1G* is the center node connecting most of the identified significant genes. **Figure 4C** is the known signaling pathway of FcER1, where the proteins including FcER1, TNF, SLP, and Ras are also present in the common 28 genes list.

### Cohort 3: One Target, FcER1, With the Most Significant Expression With DKD Severity Was Validated in an Independent Set of DKD Samples

**Figure 6:**
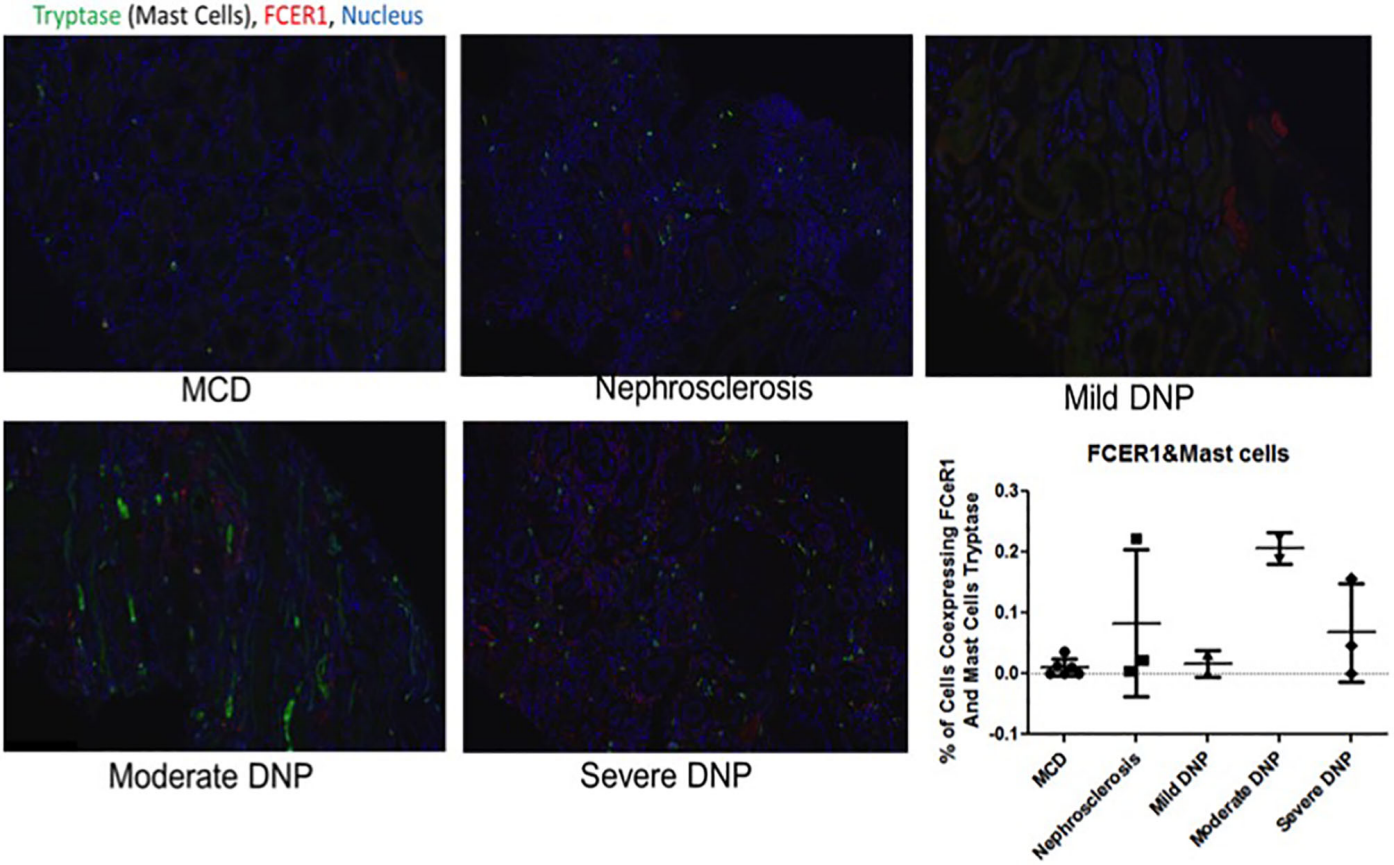
Representative immunofluorescence staining for coexpression of *FcER1* and inflammatory cell markers in human kidney tissue. Coexpression of *FcER1* and mast cell marker. MCD, Minimal changed disease; DN, diabetic nephropathy.

IHC was performed to validate the relevance and localization of FcER1 in an independent set of kidney biopsy tissue samples obtained from patients with varying CKD stages of DKD (Cohort 3). FcER1 expression was high in the renal interstitium and some in glomeruli. Significantly increased immunopositivity to FcER1 was noted in samples with DKD (**Figures 5A**–**D**), with significance for a progressive increase in staining intensity of FcER1 with disease severity [mild-moderate DN (p < 0.01) and severe DN (p < 0.001)] when compared to controls (**Figure 5E**). The quantitative changes in *FcER1* tissue gene expression in advancing DKD disease in Cohort 3, were mirrored by a similar trend in quantitation of FcER1 PBMC gene expression in advancing DKD disease, in an entirely different cohort of patients in Cohort 1 (**Figures 5F, G**). IF co-staining DKD Cohort 3 tissue samples with both FcER1 and inflammatory cell markers, specifically showed a co-expression with tryptase, a mast cells marker (**Figure 6**).

## Discussion

Current therapy for DKD is mostly focused on the use of angiotensin-converting enzyme inhibitors (ACEi) or angiotensin receptor blockers (ARBs) to reduce intra-glomerular pressure (44) along with multifactorial interventions such as improved control of blood pressure, blood glucose, and lipid, and smoking cessation as a means of slowing the atrophy of renal function in diabetes (45). Sodium-glucose-linked transporter 2 (SGLT2) inhibitors have been shown to further reduce renal events in patients with diabetes as an additive benefit over ACEi or ARB therapy. SGLT2 inhibitors suppress renal glucose reabsorption to reduce blood glucose and body weight, alter renal hemodynamics, reduce intraglomerular pressure, attenuate diabetes-associated hyperfiltration, tubular hypertrophy, and tubular toxicity of glucose to directly protect the kidney. Furthermore, SGLT2 inhibitors reduce the workload of the proximal tubules to improve tubulointerstitial hypoxia, and then allow fibroblasts to resume normal erythropoietin production, and thereby protecting the kidney (46).

While DKD is not considered a primarily “immune-mediated” form of kidney disease, extensive evidence supports the involvement of many immune system components in DKD progression and even initiation. Emerging knowledge about the dysregulation of the immune response and inflammation, suggests a key pathogenic link between aberrant innate and adaptive immunity, metabolism, and progressive kidney damage, and thus open the door to explore new immune-modulatory therapies in DKD. Inflammation is being increasingly understood to be a prominent pathological feature of diabetic nephropathy. Increased inflammation and inflammatory markers have been shown to predict worse renal outcomes (15). Recently, baricitinib and a CCR2 antagonist CCX140 have been shown to reduce proteinuria in DKD, likely because of a global effect on renal tissue inflammation (47, 48). CCR2 expressed by monocytes and macrophages are the main receptor for the pro-inflammatory chemokine CCL2. Urinary excretion of CCL2 correlates with the severity of DKD, again supporting the role of increased inflammation and renal injury in diabetes (49–52). Additionally, the importance of pharmacologic intervention to block inflammation in the diabetic kidney, can be seen in pre-clinical models, where either blockade of *CCR2*, or lack of CCR2 in a knockout model, can reduce renal macrophage and monocyte infiltration and may translate to a downstream reduction of interstitial fibrosis in the kidney (45, 53).

Recognizing that there is a critical immune-mediated component of renal injury in diabetes, we undertook an innovative study design (**Figure 1**) of unbiased transcriptional discovery and independent gene and protein validation of key dysregulated pro-inflammatory pathways in independent patients with diabetes with varying stages of DKD injury, examining different tissue sources, PBMC (**Figure 2**) and kidney tissue (**Figure 3**). This analysis reveals a skewed gene expression profile of an overlapping hub of genes, in both specific immune cells in the blood and similar infiltrating immune cells in the DKD kidneys, with significant enrichment in Fc epsilon receptor (*FcER1*), and T-cell receptor-mediated signaling pathways (**Figures 2E**, **3B**). From the list of candidate genes, *tumor necrosis factor (TNF)*, *toll-like receptor (TLR)*, and *chemokine receptor 1 (CCR1)* have been previously described in the literature as playing a role in DKD; supporting the biological relevance of the identified gene-set in this study (54–58). Microarray analysis of the PBMC dataset (Cohort 1), identified *FcER1, TRPC3, SNX20, FAM20A*, and *SLC12A7* as genes showing increased expression with the severity of diabetes (**Supplementary Table 1**). *FcER1* and *TRPC3*, both are expressed and associated with inflammatory signaling in mast cells. SNX20 plays a role in cellular vesicle trafficking, *FAM20A* is expressed in hematopoietic cells, and SLC12A7 is required for basolateral Cl (–) extrusion in the kidney and contribute to renal acidification. In Cohort 2, our eGWAS meta-analysis data evaluated up-regulated genes in microdissected glomeruli from DKD patients. Comparing both the PBMC and kidney tissue in independent DKD cohorts surprisingly identified a common set of 28 genes with significantly increased expression in both DKD PBMCs and glomerulous (**Table 2**), with high significance for *FcER1* in DKD disease (**Figure 4**). Further expression and co-localization of FcER1 in yet another independent set of human DKD kidney tissue samples, with varying stages of renal injury, confirmed increased FcER1 gene and protein levels in human DKD injury, where FcER1 expression co-localized in infiltrating mast cells (**Figures 5**, **6**). It is important for us to understand that glomerular genes in cohort 2 includes the entire glomerulus consists of glomeruli and glomerular-interstitial spaces. Tubulointerstitium is a continuation of the glomerular interstitium. So it is understandable that the inflammatory response is spilling into these continual mesenchymal spaces as the DKD pathology recruits more cells. Also, even in the case of early DKD though the major involvement is of the glomerulus, there is inflammatory cells recruitment from the tubular interstitium. All these could explain the expression of FcER1 protein in tubules as well (**Figures 5** and **6**). In future, we will address the tubular involvement in DKD and its association with *FcER1*.

*FcER1* is a high-affinity IgE receptor expressed on mast cells, basophils, eosinophils, and antigen-presenting cells (59). Mast cells are an innate immune cell type that has been previously shown to infiltrate the renal parenchyma in pre-clinical models of DKD and human diabetic kidney disease. We hypothesize that mast cells increasingly infiltrate the human diabetic kidney and support an inflammatory milieu, through increased expression of *FcER1*, which then may play a critical role in DKD progression. Increased intra-renal FcER1activation in infiltrating mast cells in a patient with diabetes may promote the release of many inflammatory mediators, including TGF-β, TNF-α, IL6, Tryptase, IL1, which can then subsequently drive the development of renal fibrosis. In diabetic nephropathy, mast cells participate in renal fibrosis by contributing to excessive accumulation of extracellular and mesangial matrix and also producing non-fibrillar short-chain type VIII collagen (60). Pre-clinical studies support this hypothesis, as mast cell inhibitors such as cromolyn or ketotifen (Zaditor) have been found to protect mice with diabetes from the development of renal injury (27). Some of this reno-protective effect may likely be mediated by reducing the expression of *FcER1*. In this study, we found coexpression of the mast cell marker tryptase and FcER1 in DKD with a trend towards higher tissue expression of both with DKD progression (**Figure 6**). This fact argues for the biological relevance of this axis in DKD damage rather than this only being a non-specific effect of the kidney being just exposed to a diabetic milieu. Also, we noted that the PBMC transcriptome of healthy and ESRD cluster closer together in **Figure 2**, which is consistent with the IF results in **Figure 6**, where the kidney FcER1 expression appears to be dampened in patients with severe diabetic nephropathy compared with moderate disease. We believe this is because both, healthy and ESRD, have lower levels of immune response genes compared to active DKD kidney injury.

In conclusion, we demonstrate how transcriptomic datasets may be combined and integrated to highlight the most robust markers. This study highlights the importance of both immune and non-immune mechanisms driving diabetic nephropathy. The innate immune response occurring in the diabetic kidney is an anticipated consequence of the chronic stresses and injury in the diabetic kidney. Ultimately, the ongoing diabetic renal inflammation results in substantial kidney damage with progressive fibrosis that eventually leads to end-stage renal disease. Therefore, therapeutic strategies targeting the innate immune system will be important for the treatment of diabetic nephropathy. This is the first study implicating a direct role of the IgE receptor FcER1 in DKD progression, thus uncovering a new druggable target for improving DKD or slowing DKD progression. Further validation studies are planned to evaluate the role of FcER1 blockade in preclinical models of diabetes to directly address the efficacy of targeting this molecule as a novel therapeutic for DKD.

## Data Availability Statement

The datasets presented in this study can be found in online repositories. The names of the repository/repositories and accession number(s) can be found below: https://www.ncbi.nlm.nih.gov/, GSE142153.

## Ethics Statement

Written informed consent was obtained from all the participants, and the study was approved by the institutional review boards of the University of Wisconsin and University of California, San Francisco

## Author Contributions

MN conducted experiments. SS, PB, and MN performed data analysis, data interpretation, manuscript writing. HS participated in the sample collection and study design. TS contributed in experiments, data analysis and manuscript writing. MS conceived the study, analysis and data interpretation, writing and revising the manuscript critically for intellectual content. All authors contributed to the article and approved the submitted version.

## Funding

This work was funded by two grants, NCATS/NIH (R21TR001761) and NIDDK/NIH (R01DK109720).

## Conflict of Interest

The authors declare that the research was conducted in the absence of any commercial or financial relationships that could be construed as a potential conflict of interest.

## Publisher’s Note

All claims expressed in this article are solely those of the authors and do not necessarily represent those of their affiliated organizations, or those of the publisher, the editors and the reviewers. Any product that may be evaluated in this article, or claim that may be made by its manufacturer, is not guaranteed or endorsed by the publisher.
