# Supplementary material for: FcER1: A Novel Molecule Implicated in the Progression of Human Diabetic Kidney Disease
Source: Front Immunol. 2021 Dec 1;12:769972. doi: 10.3389/fimmu.2021.769972 (PMC8672419; doi:10.3389/fimmu.2021.769972)
Supplement: Supplementary file 1 [file Table_1.docx]

**SUPPLEMENTARY TABLE 1:**

| **GENE_SYMBOL** | **D1 (antilog)** | **D2 (antilog)** | **D3 (antilog)** | **p value** |
| --- | --- | --- | --- | --- |
| *FCER1G* | 0.385 | 0.734 | 1.220 | 0.00012 |
| *TRPC3* | 0.947 | 1.666 | 1.903 | 0.00055 |
| *SNX20* | 1.130 | 1.858 | 2.963 | 0.00071 |
| *FAM20A* | 0.729 | 1.388 | 2.516 | 0.00114 |
| *SLC12A7* | 0.435 | 0.642 | 1.772 | 0.00114 |
| *FLVCR1* | 0.761 | 1.724 | 1.764 | 0.00144 |
| *MTHFR* | 0.531 | 0.904 | 2.484 | 0.00144 |
| *TECTA* | 0.932 | 1.325 | 1.829 | 0.00180 |
| *AHNAK2* | 0.777 | 1.308 | 1.664 | 0.00180 |
| *C1orf125* | 0.983 | 1.287 | 1.582 | 0.00180 |
| *TMED5* | 0.955 | 1.123 | 1.167 | 0.00225 |
| *GUSB* | 0.898 | 1.105 | 1.264 | 0.00225 |
| *ELMO2* | 1.041 | 1.308 | 2.358 | 0.00225 |
| *ISCU* | 0.747 | 1.234 | 1.502 | 0.00280 |
| *ZIC1* | 0.858 | 1.000 | 1.713 | 0.00280 |
| *NOM1* | 1.051 | 1.239 | 1.971 | 0.00280 |
| *RAB35* | 1.079 | 2.070 | 2.722 | 0.00280 |
| *ARHGEF10* | 0.483 | 0.783 | 1.011 | 0.00280 |
| *OTOF* | 0.809 | 0.952 | 1.343 | 0.00280 |
| *TMEM87A* | 1.296 | 1.870 | 2.059 | 0.00280 |
| *ITGAX* | 0.880 | 0.994 | 3.510 | 0.00280 |
| *LCP2* | 0.700 | 0.989 | 1.103 | 0.00346 |
| *C10orf82* | 0.883 | 1.192 | 1.893 | 0.00346 |
| *MTMR14* | 0.712 | 0.927 | 1.422 | 0.00346 |
| *RBPJ* | 0.771 | 1.380 | 1.760 | 0.00426 |
| *JAG2* | 1.278 | 2.212 | 3.863 | 0.00426 |
| *PDGFRB* | 1.212 | 1.768 | 8.561 | 0.00426 |
| *IPMK* | 0.686 | 1.763 | 1.396 | 0.00426 |
| *CPEB4* | 1.253 | 2.967 | 2.529 | 0.00426 |
| *PPP6C* | 1.077 | 2.073 | 2.125 | 0.00523 |

**Supplementary Table 1**. Top 30 significant genes based on JT test for increasing expression with diabetes severity. Anti-logged median expression and p value from JT test shown. Diabetes with normoalbuminuria, D1; diabetes with microalbuminuria, D2; diabetes with macroalbuminuria, D3.
